# Development of a prognostic nomogram for sepsis associated-acute respiratory failure patients on 30-day mortality in intensive care units: a retrospective cohort study

**DOI:** 10.1186/s12890-022-02302-6

**Published:** 2023-01-30

**Authors:** Mengdi Luo, Qing He

**Affiliations:** Southwest Jiaotong University of Medicine/Southwest Jiaotong University Affiliated Chengdu Third People’s Hospital, Chengdu, 610031 Sichuan China

**Keywords:** Sepsis, Sepsis associated-acute respiratory failure, Nomogram, Intensive care unit

## Abstract

**Background:**

Acute respiratory failure is a type of sepsis complicated by severe organ failure. We have developed a new nomogram for predicting the 30-day risk of death in patients through a retrospective study.

**Method:**

Data was collected and extracted from MIMICIV, with 768 eligible cases randomly assigned to the primary cohort (540) and the validation cohort (228). The final six factors were included by Cox regression analysis to create the Nomogram, the accuracy of the Nomogram was assessed using the C-index and calibration curve, and finally, the clinical usefulness of the Nomogram was evaluated using DCA in.

**Results:**

Multivariate Cox regression analysis showed that age, DBP, lactate, PaO2, platelet, mechanical ventilation were independent factors for 30-day mortality of SA-ARF. The nomogram established based on the six factors. The C-index of nomogram in the primary cohort is 0.731 (95% CI 0.657–0.724) and 0.722 (95%CI 0.622–0.759) in the validation cohort. Besides, the decision curve analysis (DCA) confirmed the clinical usefulness of the nomogram.

**Conclusion:**

The study developed and validated a risk prediction model for SA-ARF patients that can help clinicians reasonably determine disease risk and further confirm its clinical utility using internal validation.

## Introduction

Sepsis is a lethal syndrome of physiologic, pathologic, and biochemical abnormalities induced by infection, one of the major global public health concerns [1]. Although extensive research has recently demonstrated the mechanism and treatment of sepsis, sepsis is still the principal cause of death in intensive care patients worldwide [2]. For the evaluation of organ dysfunction or failure, there is currently the Sequential Organ Failure Assessment (SOFA) or Quick Sequential Organ Failure Assessment (qSOFA), which does not include pulmonary function other than with a respiratory rate [3]. Patients might be impacted with acute respiratory failure (ARF) if they fulfilled either diagnostic criteria. Notably, early diagnosis and treatment can help reduce mortality.

Therefore, acute respiratory failure was the common sepsis-associated organ injury resulting in critical mortality [4–6]. ARF is due to severe dysfunction of pulmonary ventilation. Moreover, a decrease in the arterial partial pressure of oxygen (PaO2) is a sign of pulmonary dysfunction in patients with sepsis [7]. According to accumulating studies, acute respiratory failure had a 60% mortality rate in the ICU, an average hospital stay is 7.1 days, and related medical costs are up to US$54 billion annually in the USA [8, 9]. Clinical risk factors, pathobiology, response to treatment, and elements of pulmonary recovery have been extensively studied, which has improved the prevention, detection, and treatment of acute respiratory with sepsis [10, 11].

Nevertheless, the pathogenesis of sepsis-related acute respiratory failure is still in an ongoing phase of exploration, and the different sources of risk factors make it a significant clinical challenge for early detection [12]. In recent studies, the disease characteristics of SA-ARF patients have been used to identify increased risks. However, most of them have not combined these with clinical prediction models [13, 14]. Nevertheless, few studies have focused on patients with sepsis complicated by ARF. Thus, the purpose of this study by an extensive clinical database is to evaluate the impact of SA-ARF on the 30-day mortality and to develop a predictive nomogram for predicting the probability of 30-day mortality in patients with SA-ARF.

## Method

### Data source

This retrospective study was based on the MIMIC IV (V1.0) database, a large intensive care database. Which contains comprehensive information for each patient while they were in the hospital: laboratory measurements, medications administered, vital signs documented, and so on. It is intended to support a wide variety of research in healthcare [15]. MIMIC-IV builds upon the success of MIMIC-III and incorporates numerous improvements over MIMIC-III. Informed consent for this study has been waived by the Massachusetts Institute of Technology and Beth Israel Deaconess Medical Center. The author has the right to use and download the database through the Protecting Human Research Participants exam.

### Study population

**Fig. 1:**
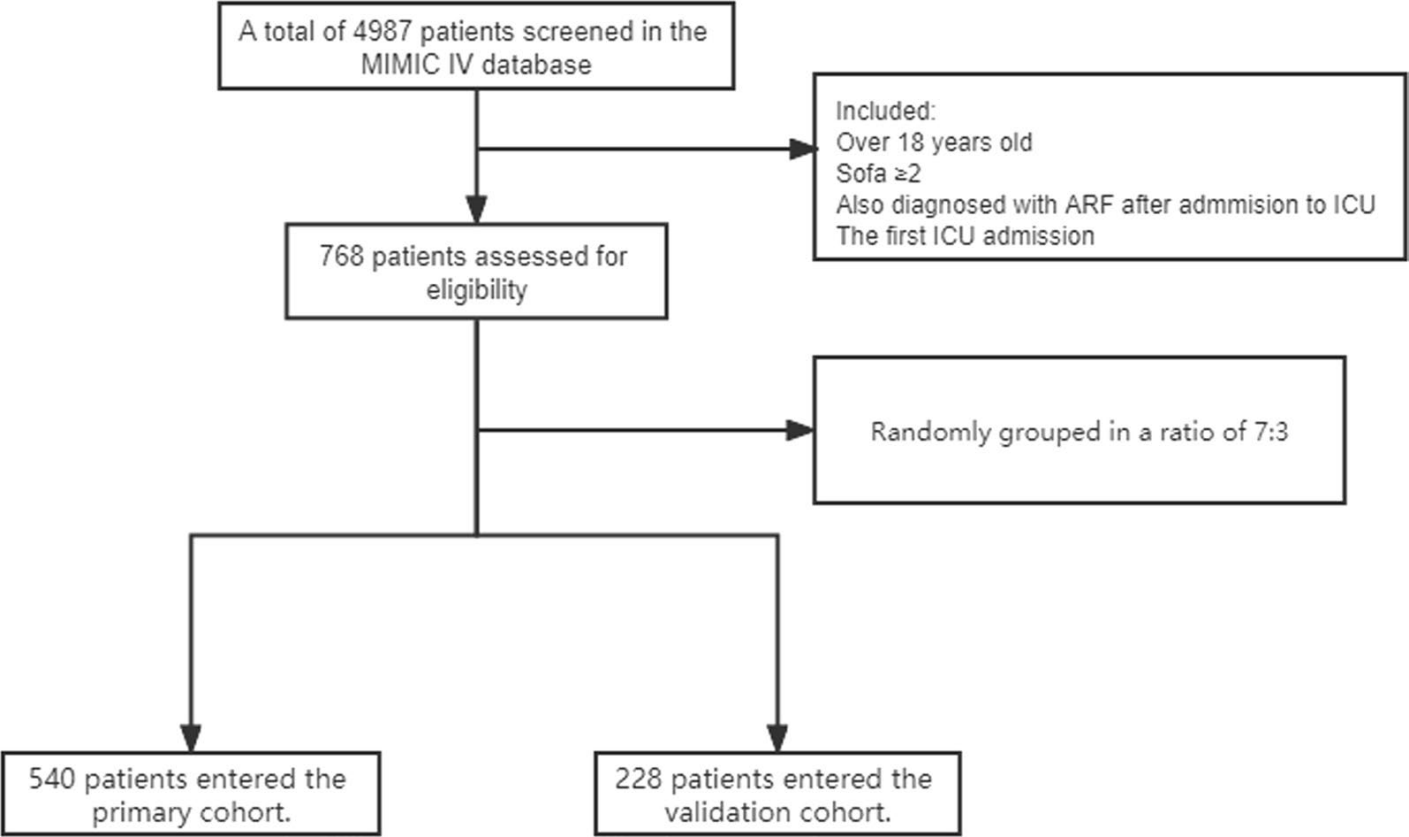
Flow chart of the study

We included patients aged > 18 years with a diagnosis of sepsis according to the International Classification of Diseases, Ninth Revision (ICD-9). Also, the MIMIC-IV database was used to identify patients with ARF based on their ICD-9 code. For patients with multiple ICU admissions, only the first ICU admission for each patient was analyzed. Patients with ARF before admission to the ICU were excluded. The cohort is randomly split into the primary cohort and the verification cohort according to the ratio of 7:3 (Fig. 1). The primary cohort is used to establish the nomogram, and the verification queue is used for verification.

### Data extraction

PgAdmin4 has used run structure query language (SQL) and extracted data from the MIMIC IV database. The following variables were extracted or calculated: (1) basic demographics including age, gender and private insurance, (2) vital signs, including heart rate, mean arterial pressure (MAP), systolic blood pressure (SBP), diastolic blood pressure (DBP) and (3) laboratory parameters, including white blood cell, hemoglobin, platelet, albumin, bilirubin, potassium, sodium, bicarbonate, lactate, creatinine, blood urea nitrogen, partial pressure of oxygen, etc. severity score, mechanical ventilation, length of ICU, hospital stay, and date of death, etc. All the data were extracted from the data generated within the first 24 h after the patient entered the ICU (i.e., the baseline value). All the scripts used to calculate the SOFA and SAPSSII scores were available from the GitHub website (https://github.com/MIT-LCP/mimic-code/tree/main/mimic-iv/concepts).

### Statistical analysis

Continuous variables were presented as median, and categorical variables were presented as a number. Categorical variables between the groups were compared using the X2 test and Fisher exact test. The object of this study was to develop a prognostic model for 30-day mortality of patients in ARF with sepsis in the ICU. Therefore, we used the Cox proportional hazard model. Univariate Cox regression was used to analyze the relationships of relevant variables with these patients. Variables with *P* < 0.05 in univariate analysis were further included in the multivariate Cox proportional hazard model. The HR, which was used to approximate the risk of an event, was also calculated. The backward stepwise process based on the Akaike information criterion was used to control the overfitting of the model.

Establish a nomogram model based on the above analysis results. Moreover, the C-index and calibration are often considered the essential property of a model. Normally, the C-index used to determine a nomogram's discrimination, which was 0.5, would not have an event. If the model always produces a higher probability of patients having events than those not, the C-index is 1.0 [16]. The calibration curve is often considered an essential property of a model and reflects the extent to which a model correctly estimates the absolute risk. Poorly calibrated models will underestimate or overestimate the outcome of interest [17]. Decision curve was a new method to evaluate the prognostic nomogram by quantifying the standardized net benefits at different threshold probabilities. In our study, a *P* value < 0.05 was considered statistically significant. All analyses were performed using Stata 16 and R software (R v4.1.1).

## Results

### Population and baseline characteristics

A total of 2057 patients with sepsis 3.0 and 768 patients positively tested for SA-ARF in the MIMIC IV database were included which have complete data within 24 h after ICU admission. The mean age of patients was 68 years old and males accounted for 56.51%. Patients were randomly devided to primary cohort (540 patients) and validation cohort (228 patients). There were no significant differences in baseline characteristics between the two cohorts (*P* > 0.05). All baseline characteristics are summarized in Table 1.

**Table 1:** Patients’ baseline characteristics in the primary cohort and validation cohort

Variables	Primary cohort (n = 540)	Validation cohort (n = 228)
*Demographic characteristics*		
Age, years	68 (58.71)	67 (56.81)
Gender, n (%)		
Male	298 (55.19)	136 (59.65)
Female	242 (44.81)	92 (40.35)
Mechanical ventilation, n (%)	342 (63.33)	126 (55.26)
Comorbidities, n (%)		
Hypertension	6 (1.11)	1 (0.44)
Diabetes	3 (0.56)	1 (0.44)
Chronic heart failure	18 (3.33)	8 (3.51)
Chronic liver disease	1 (0.19)	0
Laboratory parameters, [median, (IQR)]		
White blood cell (10^9^/L)	15.19 (11.18)	14.65 (10.18)
Hemoglobin (g/dL)	9.96 (9.10)	10.08 (8.10)
Platelet (10^9^/L)	195 (116.262)	200 (106.161)
Bilirubin (umol/L)	2.55 (1.3)	2.56 (0.2)
Albumin (g/dL(	3.14 (3.4)	3.23 (3.4)
Potassium (mmol/L)	4.25 (4.5)	4.23 (4.5)
Sodium (mmol/L)	139 (136.142)	139 (135.142)
Bicarbonate (mmol/L)	20.84 (16.25)	21.67 (18.25)
Lactate (mmol/L)	2.94 (1.3)	2.81 (1.3)
Creatinine (mg/dL)	2.05 (1.3)	1.76 (1.2)
BUN (mg/dL)	40.42 (19.54)	35.14 (19.45)
SaO2	94 (93.97)	96 (95.97)
PaO2	145 (77.166)	139 (79.166)
Vital signs		
Heart rates (/min)	95 (78.120)	93 (73.116)
DBP (mmHg)	59 (53.63)	58 (54.62)
SBP (mmHg)	114 (102.121)	117 (108.123)
MAP (mmHg)	70.92 (64.83)	72 (61.82)
Severity scores		
SOFA	5 (3.8)	6 (3.8)
SAPSII	51 (40.60)	49 (38.59)

*Bun* blood urea nitrogen, *SpO2* percutaneous oxygen saturation, *MAP* mean arterial pressure, *PaO2* alveolar oxygen partial pressure, *HR* heart rate, *DBP* diastolic blood pressure, *SBP* systolic blood pressure, *SOFA* sequential organ failure assessment, *SAPS II* simplified acute physiology score II

### Prognostic factors in the primary cohort

By applying the COX regression model to univariate prognostic analysis, a stepwise regression approach was used. Variables, including age, lactate, bicarbonate, bilirubin, heart rate, platelets, Pao2 level, DBP, chronic heart failure and mechanical ventilation, were potential predictors of 30-day mortality in the univariate analysis (*p* < 0.05) (Table 2). After adjusting for these variables, all these candidate factors were included the multivariable Cox proportional hazard model. At last, six factors were included in the final prediction model (*P* < 0.05) (Table 3).

**Table 2:** Univariate Cox regression analysis of variable to patients in primary cohort

Variables	HR	95%CI		*P*
Age	1.020	1.021	1.028	< 0.001***
HR	1.004	1.000	1.007	0.045*
MAP	0.998	0.995	1.001	0.288
DBP	0.987	0.979	0.995	0.003**
SBP	0.997	0.993	1.002	0.277
Potassium	1.006	0.998	1.025	0.498
Wbc	0.991	0.976	1.006	0.256
K	0.987	0.881	1.107	0.830
Bun	0.998	0.995	1.002	0.581
Bicarbonate	0.983	0.962	1.005	0.125
Scr	0.975	0.906	1.490	0.505
Albumin	1.050	0.934	1.180	0.409
Hemoglobin	0.983	0.919	1.053	0.639
Platelet	0.998	0.997	0.999	0.018*
Bilirubin	1.030	1.009	1.052	0.004**
Lactate	1.098	1.051	1.148	< 0.001***
PaO2	0.999	0.998	1.000	0.031*
SOFA	0.977	0.942	1010	0.169
SAPSII	1.010	1.003	1.016	0.005**
Diabetes	0.984	0.137	7.320	0.987
Hypertension	0.830	0.206	3.344	0.794
Chronic heart failure	2.279	1.349	3.850	0.002**
Mechanical ventilation	0.209	0.158	0.276	< 0.001***

**Table 3:** Results of the forward stepwise Cox regression analysis of patients in primary cohort

Variables	HR	95%CI		*P*
Age	1.010	1.005	1.022	< 0.001***
DBP	0.990	0.981	0.999	0.030*
HR	1.000	0.998	1.007	0.148
Plt	0.998	0.997	1.000	0.045*
spassii	1.003	0.995	1.010	0.435
Bilirubin	1.019	0.997	1.042	0.084
Lactate	1.108	1.054	1.165	< 0.001***
Pao2	0.998	0.997	0.999	0.003**
CHF	1.168	0.986	2.893	0.055
Mechanical ventilation	0.217	0.163	0.289	< 0.001***

### Development of a prediction nomogram

**Fig. 2:**
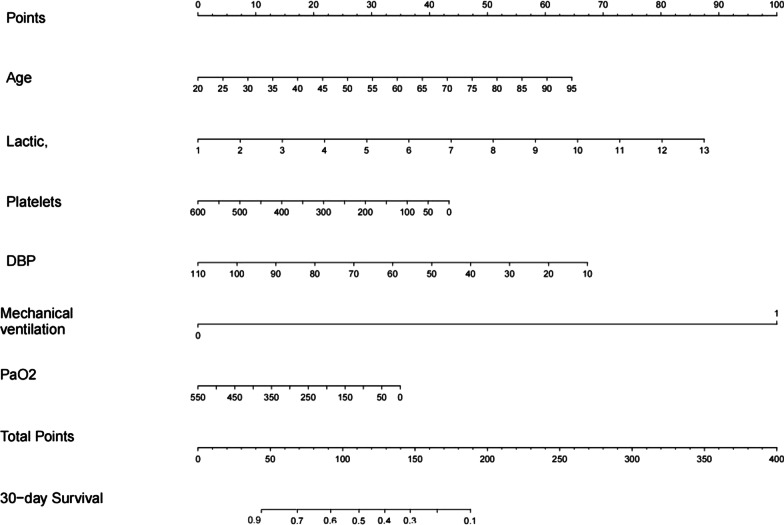
Nomogram for calculating risk scores and predicting the 30-day survival probability of SA-ARF patients. To use this, a vertical line was drawn upwards from each variable and the corresponding score was recorded ("age = 55" = 30 points). Finally, all scores were summed and the vertical projection from the total dotted line down to the 30-day survival line was equivalent to predicting the 30-day survival probability for SA-ARF patients. Abbreviations: *DBP* diastolic blood pressure

A nomogram for predicting the 30-day survival was constructed with the six variables that had most significant values in multivariate Cox proportional hazards regression (Fig. 2).

The nomogram was established to standardize the regression coefficients (HR values), and then display them as risk scores on the number line. To use the nomogram, first judge the scores for each factor, then add up the total scores and finally draw a vertical line from the total points line down to the straight 30-day survival line. This gives an estimate of the probability of 30-day survival.

For instance, a patient with sepsis and respiratory failure was 55 years old (30 points), a lactate level of 3 mmol/L (15 points),without ventilation, platelet with 50 K/uL (40 points), DBP with 80 mmHg (20 points), Pao2 level of 150 (25 points), had a total score of 130 points and the 30-day survival probability was 40%.

### Validation of the prediction nomogram

**Fig. 3:**
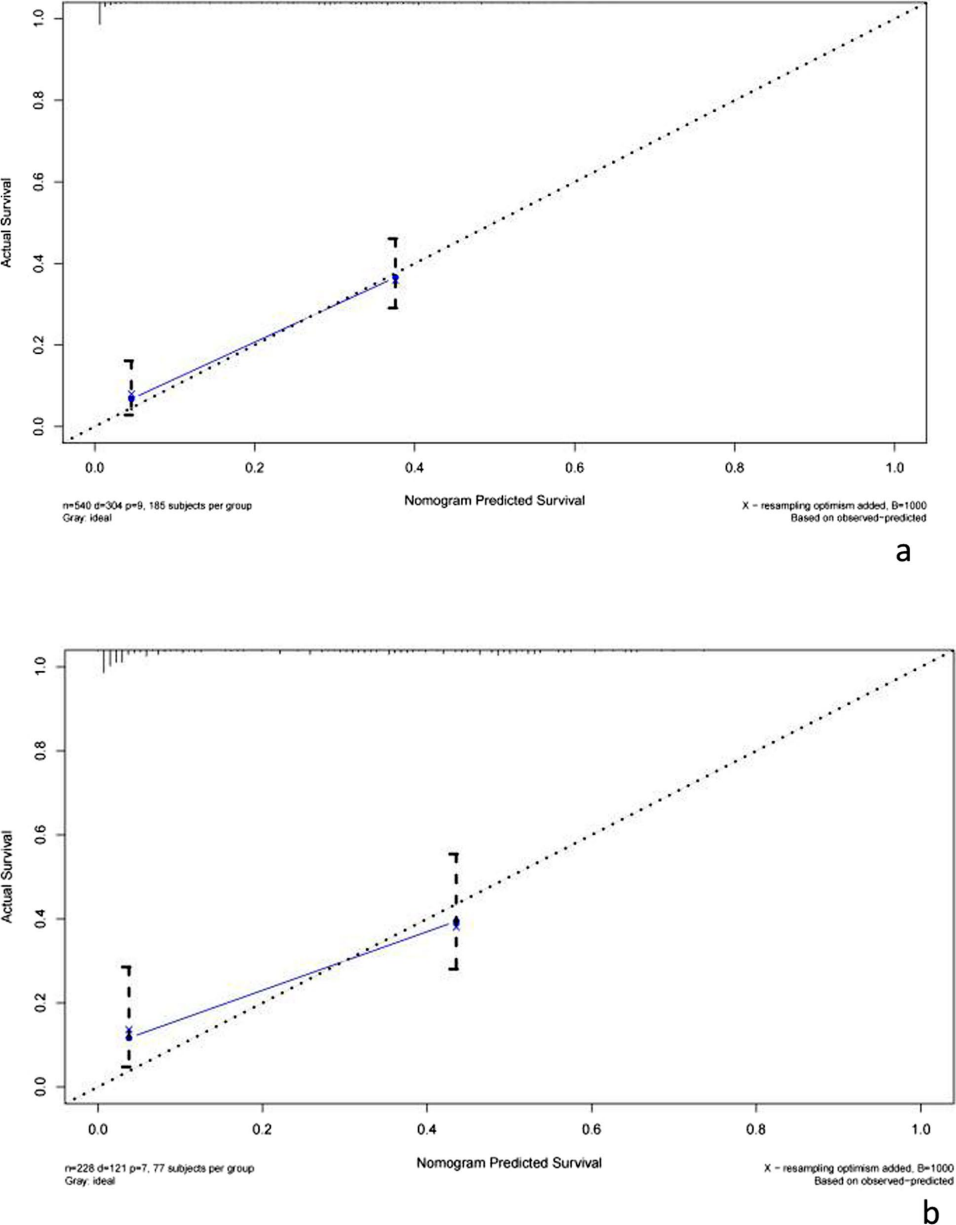
Calibration curves for nomogram in the primary (**a**) and validation (**b**) cohorts

Using the 1000 bootstrap resampling method, the performance of the prediction nomogram was internally validated in the validation set. The calibration curve of the nomogram for the prediction of 30-day survival in sepsis patients demonstrated good agreement both in the primary cohort and validation cohort (Fig. 3a, b). Moreover, the C-index for the prediction nomogram was 0.731 (95% CI 0.657–0.724) for the primary cohort and 0.722 (95%CI 0.622–0.759) for the validation cohort.

### Clinical use of the nomogram

**Fig. 4:**
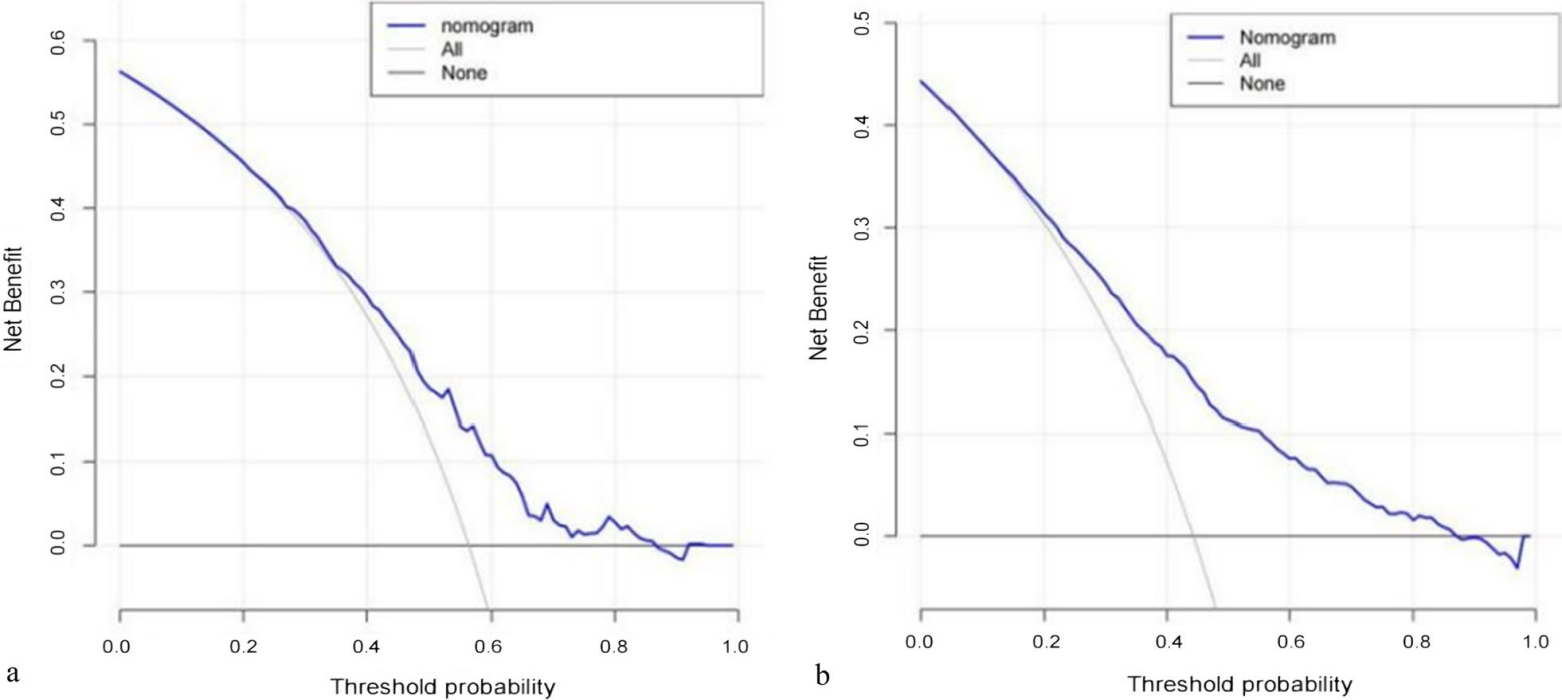
Decision curve analysis for nomogram in the he primary (**a**) and validation (**b**) cohorts

Finally, the decision curve analysis (DCA) was used to confirm the findings. The analysis of the decision curve showed that in the primary cohort and in the validation cohort, the threshold probability of our nomogram is 0.92 in the primary cohort and 0.85 in the validation cohort (Fig. 4a, b). Within this range, the predictive effect of the nomogram is better than that of a single predictor, respectively.

## Discussion

This retrospective analysis study extracted clinical data from an extensive database named MIMIC IV, which has more than 500,000 medical records from 2008 to 2019. Using Cox regression models, we conducted the risk factors related to 30-day mortality of ARF with sepsis, which included age, DBP, lactate, bilirubin, Pao2, SAPS II, CHF, and the use of mechanical ventilation. We established a prognostic nomogram for these patients in the ICU. The nomogram can be used to predict and diagnose. As far as we know, this is the first study to evaluate the risk factors associated with 30-day mortality about ARF with sepsis in the ICU and establish a nomogram.

An inflammatory response, immune suppression, and oxidative stress led to sepsis. Moreover, the effects of an inappropriate response to this infection resulted in impaired cellular function, mitochondrial dysfunction, and ultimately acute respiratory failure [18]. Although the mechanisms by which sepsis leads to respiratory failure are still unclear, the development of these mechanisms can be balanced with the variables in our model [19, 20]. Therefore, this model may have important implications for the development of acute respiratory failure in sepsis.

Acute respiratory failure is a common complication in patients with sepsis. Early diagnosis of ARF is usually confirmed by clinical manifestations, radiographs, CT, and pulmonary function, which are cumbersome operational steps [21]. In recent years ultrasound technology has also played an essential role in diagnosing ARF patients in the ICU [22]. What is more, sepsis is usually diagnosed by SOFA score or qsofa score [23]. However, few studies have mentioned association between ARF and short-term mortality in sepsis. Based on this, it is vital for clinicians to conduct a thorough evaluation of the risk of death from sepsis and to objectively estimate the risks and benefits of medical interventions so that patients and families can make medical decisions with a careful assessment of the impact of potential treatment options. This will not only prevent medical disputes but also reduce certain medical costs. Thus, prediction nomograms are crucial for improving the risk stratification process of sepsis and can be used by clinicians to provide clear, accurate information to families of patients with SA-ARF. Therefore our nomogram used six factors that were easily accessible and could be collected on the first day of admission. We hope that this chart will enable us to identify sepsis with acute respiratory failure quickly.

Our study's initial vital signs include diastolic blood pressured and PaO2, which were considered an independent risk factor for patients with acute respiratory failure in SA-ARFs, the same result as the previous study [24]. Age was widely recognized as the most powerful risk factor for organ failure in ICU. Increasing age was a major determinant of organ failure and overall mortality [25, 26]. A large cohort study proved that patients who died with sepsis also tended to be older adults [27]. In summary, age is strongly associated with poor prognosis in patients with acute respiratory failure in sepsis. Lactate was more dominant in the nomogram, which was the most significant predictor of mortality at 30 days in patients with sepsis-associated acute respiratory failure. Lactate levels can indicate the severity of the underlying disease. Moreover, it has been shown that elevated lactate levels may predict death in critically ill patients, while reduced lactate levels have been reported to be associated with improved clinical outcomes [28, 29]. Jean-Louis Vincent et al. indicated that lactate levels are associated with prognosis in sepsis and acute respiratory failure patients [30, 31]. Consistently, our study found that lactate is an Independent predictor for 30-day mortality of SA-ARF. In one sense, sepsis can be seen as a race to the death between the pathogens and the host immune system. The exaggerated response can lead to multi-organ failure (MOF), especially respiratory failure. As we all know, the interaction of platelets with immune cells and endothelial cells is an anti-infective response.

Nevertheless, during sepsis, the mechanisms become dysregulated and contribute to organ damage [32]. One experiment proved that thrombocytopenia exacerbated the inflammatory response to sepsis and increased mortality [33]. These experimental results are consistent with the results of our study. Besides, during sepsis, most patients require ventilation therapy. According to a randomized clinical trial, mechanical ventilation and conservative oxygen therapy are associated with poor outcomes in patients with sepsis [34]. In short, these six factors are all predictors of mortality in respiratory failure. They are all clinically accessible nomograms that have been widely used in cancer research, and are increasingly being utilized in the prediction of diseases [35, 36]. The nomogram was developed to survive acute respiratory failure in the elderly at 28 days, 60 days, and one year [37]. For the mortality of SA-ARF, our study is the first to propose a simple nomogram to predict its mortality. The six included predictors were readily available on the first day of ICU admission. Furthermore, following calibration by internal validation to the actual 30-day mortality, we found high agreement between the training set and the validation set. In this cohort, the C-index was more than 0.7, and the calibration analysis performed in two cohorts revealed that the predicted 30-day mortality was similar. In addition, all decision curves in the cohort have net benefit rates above 50 percent.

Furthermore, there are two points to consider when using the nomogram. In our study, vital signs are calculated based on the average of each ICU patient's first 24 h in the ICU. Accordingly, the nomogram is not relevant to patients who die within 24 h of admission or who leave the intensive care unit within 24 h. In addition, laboratory tests included in the nomogram are the first results to be obtained from the ICU; consequently, all the laboratory tests included in the nomogram should be finished within 24 h of admission to the ICU.

The paper has some limitations despite these inspiring findings. First, this retrospective study returned the necessary recall bias, so more prospective studies need to be done to confirm this further. Second, since the values for vital signs and laboratory tests are for the first 24 h of admission to the ICU, the model does not apply to patients who die within 24 h of admission to the ICU. Third, with regard to data processing, we discarded variables with missing values greater than 40%; the retained variables were then supplemented by means of interpolation, multiple interpolation, and model predictions. In this way, the results of the analysis may have been biased, and the C-index may have been less accurate. Finally, we have only performed internal validation, so a larger sample size is needed to demonstrate its feasibility.

## Conclusion

With our nomogram based on the patients' age, lactate, platelets, DBP, Pao2 together with mechanical ventilation, we can predict 30-day mortality in SA-ARF easily. Which helps clinicians make risk based decisions and treatment strategies. However, we need further validation studies on larger sample size and the noninclusion of patients discharged from ICU before 24 h.

## Data Availability

All data generated or analyzed during this study are included in this published article.

## Abbreviations

ST-ARF: Sepsis associated-acute respiratory failure
ARF: Acute respiratory failure
MIMIC-IV: Medical Information Mart for Intensive Care IV
BIDMC: Beth Israel Deaconess Medical Center
ICU: Intensive care units
DCA: Decision curve analysis
DBP: Diastolic blood pressure
PaO2: Alveolar oxygen partial pressure
SOFA: Sequential organ failure assessment
qSOFA: Quick sequential organ failure assessment score
SAPS II: Simplified acute physiology score II
HR: Hazard rate
MAP: Mean arterial pressure
SBP: Systolic blood pressure
PaO2: Alveolar oxygen partial pressure
SpO2: Percutaneous oxygen saturation
Bun: Blood urea nitrogen
WBC: White blood cell
Scr: Serum creatinine
CHF: Chronic heart failure
